# Uptake and release characteristics of serotonin hydrochloride by natural Cuban zeolite containing clinoptilolite and mordenite

**DOI:** 10.1038/s41598-021-93487-z

**Published:** 2021-07-12

**Authors:** Jan-Paul Grass, Ulrike Pals, Alexandra Inayat, Wilhelm Schwieger, Martin Hartmann, Wilfried Dathe

**Affiliations:** 1 Institute of Chemical Reaction Engineering, Friedrich-Alexander-Universität Erlangen-Nürnberg, Egerlandstraße 3, 91058 Erlangen, Germany; 2 Center for Interface Research and Catalysis (ECRC), Friedrich-Alexander-Universität Erlangen-Nürnberg, Erlangen, Egerlandstraße 3, 91058 Erlangen, Germany; 3 Heck Bio-Pharma GmbH, Gerberstraße 15, 73650 Winterbach, Germany

**Keywords:** Biochemistry, Drug discovery, Biogeochemistry, Gastroenterology, Medical research

## Abstract

Serotonin (5-HT) plays an important role in human physiology. An excess of this native regulator within the human gut can be partially controlled by orally consuming zeolite. Therefore, this study focuses on the kinetics of the uptake and release of serotonin hydrochloride (5-HT-hc) by natural Cuban zeolite containing clinoptilolite and mordenite at different pH levels using UV–Vis spectroscopy. 5-HT-hc is stable under the following investigated experimental conditions: incubation temperature of 36 °C; and at a pH of 5, 7, and 9. Independent of the zeolite framework, the 5-HT-hc is adsorbed without changing its molecular structure. The uptake and release of 5-HT-hc were not correlated to the textural properties of these aluminosilicates. The investigated zeolites adsorbed 5-HT-hc at about 14 mg per gram zeolite with no large differences observed between different samples. Release studies of 5-HT-hc-loaded zeolite revealed that the 5-HT-hc is strongly bound to the zeolite, and independent of the pH value and zeolite framework only up to 12.7% was released into the water.

## Introduction

Serotonin or 5-hydroxytryptamine (5-HT) is not only known as a feel-good hormone, but also has numerous physiological functions within the human body^1^. Among them, 5-HT is involved in triggering and maintaining the sleep–wake cycle^2^. Furthermore, serotonergic dysregulation is linked to sleep disorders and the development of Parkinson’s disease^3^. Moreover, stress-induced perturbation of the 5-HT system disrupts the circadian rhythm and increases susceptibility to depression^4^. Notably, it is vital to study the central 5-HT system’s multifaceted role in the maternal brain to understand the processes that govern matrescence and the maintenance of motherhood^5^. Therefore, an adequate endogenous level of 5-HT influences many physiological and psychological parameters, thereby eventually deciding about everyone’s mood and well-being^1^.

In contrast, an overproduction of 5-HT results in a disease pattern of neuroendocrine tumors (NETs)—called the “carcinoid syndrome”—which involves severe diarrhea and flushing as well as some other health complaints^6^. Using synthetic analogues of somatostatin is the established treatment to control these symptoms, which are at least partially caused by an overproduction of 5-HT^7^. However, some patients are not satisfied with a lower living quality caused by an insufficient reduction in bowel movements resulting from this treatment to downregulate 5-HT in the gastrointestinal tract. Recently, a case study was published showing that the oral application of a natural zeolite formulation called DETOXSAN was able to reduce bowel movements in approximately 70% of patients suffering from therapy-refractory diarrhea^8^. Therefore, it is especially interesting to investigate the ability of natural zeolites in binding 5-HT to not only remove overproduced 5-HT from the body, but also to analyze its uptake under normal physiological conditions. This is particularly important because DETOXSAN is used as medical advice.

The largest amount of 5-HT is synthesized within the mucosa of the gut wall in enterochromaffin cells. Although it is not required for the generation of neurogenic motor patterns like peristalsis, it is possible that an upregulation of serotonergic signaling pathways may change bowel motility during intestinal inflammation^9^. Therefore, detailed knowledge about the uptake properties of the zeolite with respect to 5-HT may help elucidate its therapeutic effect in carcinoid patients^8^ and serve as an alternative route to develop therapeutic agents. Consequently, this study focuses on investigating the uptake and release of 5-HT-hc by natural Cuban zeolite as found in the formulation DETOXSAN under weak acid and neutral pH conditions, which exist in the intestine. In order to understand the adsorption mechanism, basic pH conditions were also studied. 5-HT was used as a hydrochloride (hc) because free 5-HT is stored outside living matter at 4 °C and not very stable at room temperature^10^.

Zeolites are microporous, crystalline aluminosilicates that are employed in adsorption, ion-exchange, and molecular sieving. Therefore, they are often used also in environmental remediation, biotechnology, and medicine. In the field of health protection, the concentration of pesticides and organic pollutants in the environment is reduced mainly by using synthetic and modified zeolites that exhibit a high adsorption capacity for these harmful substances^11,12^; however, only natural zeolites, mainly clinoptilolite (HEU), have been used in all clinical applications. Wider medical applications of these zeolites include the exchange of cations against heavy metals, consolidation and stabilization of the immune system, reduction of discomfort within the gastrointestinal tract during heartburn and gastritis, and maintenance of body homeostasis and good health status as summarized in several reviews^13–16^. Natural zeolites modified with bi-layered surfactants have also been used in drug research to determine the adsorption capacity for anti-inflammatory drugs, such as ibuprofen and naproxen from water^17^. Notably, the bi-layered surfactant modified HEU adsorbs these non-steroidal drugs with the same range of magnitude (between 15 and 20 mg per g of zeolite) as that reported for the inflammation promotor, histamine, by the unmodified natural Cuban zeolite depending on particle size and the pH of the solution^18,19^. Moreover, natural Cuban zeolite shows antiphlogistic effects as determined by a murine inflammation model^20^. DETOXSAN powder is a zeolite formulation that is highly active in combating diarrhea^8^; DETOXSAN paste restores skin inflamed by mycosis and intertrigo^21^ and relieves other health complaints as summarized recently^22^. We only investigated natural Cuban zeolite in detail because of our interest in its medical applications.

## Results and discussion

### Chemical and textural properties of the zeolites

The chemical and textural properties of the active ingredient of DETOXSAN, natural zeolite from Cuba, have been described by Selvam et al. in detail and are briefly summarized here to compare its properties with the pure constituents, HEU and Mordenite (MOR) that were used as reference zeolites. The Cuban zeolite was scanned using electron microscopy and the absence of fibrous particles was observed, which is a prerequisite for its medical use^18,19^.

**Figure 1:**
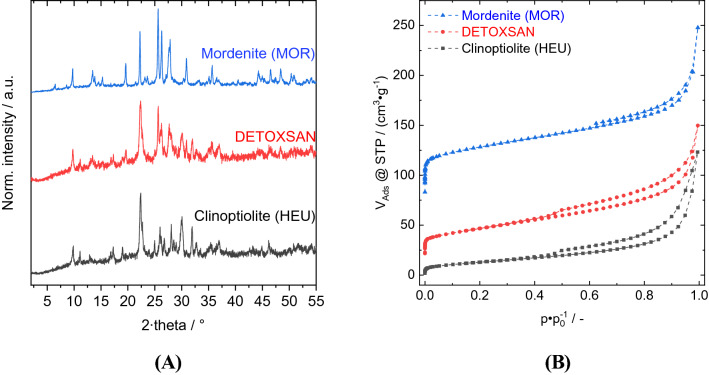
X-ray diffraction patterns (**A**) and nitrogen physisorption isotherms (**B**) for mordenite, clinoptilolite, and DETOXSAN, a mixture of 43% HEU and 35% MOR. (**A**) Shows the normalized intensity (norm. intensity) of the X-ray beam in dependence of the diffraction angel (2 theta). (**B**) Shows the adsorbed nitrogen volume at standard temperature and pressure (V_ads_@STP) as a function of the relative pressure (p p_0_^−1^).

Figure 1 shows that DETOXSAN contains a mixture of 43% HEU and 35% MOR^19^. According to previous studies^18^, the remaining 22% are unidentified amorphous materials. This quantification using crystallinity is based on the diffraction patterns of pure HEU and MOR, which are shown in Fig. 1A.

The purity of the zeolites was confirmed by comparing them with simulated reference patterns of the ICSD (Inorganic Crystal Structure Database). Figures S1 and S2 show the simulated patterns as well as their corresponding ICSD codes. All reflections can be attributed to the respective zeolite structure.

The molar composition was determined using inductively coupled plasma—optical emission spectrometry (ICP-OES) (Table 1). The main charge-compensating cations in DETOXSAN are calcium, potassium, and sodium. As previously described^18,20^, a small number of other cations also reside inside the zeolite micropores. Owing to their low concentration, these cations have only a minor influence on the overall zeolite properties that are relevant to this study; therefore, they were neglected. These cations compensate for the negative charges of the aluminium ions within the HEU and MOR framework of DETOXSAN.

**Table 1:** Molar composition of the zeolite samples in relation to aluminium as well as the sum of charge-compensating cations (cc).

Zeolite	Source	Al:	Si:	Na:	K:	Ca:	Σ cc
Mordenite (MOR)	Synthetic	1.0	7.34	0.89			0.89
Clinoptilolite (HEU)	Natural	1.0	5.67	0.07	0.23	0.18	0.66
DETOXSAN (HEU/MOR)	Natural	1.0	6.58	0.22	0.16	0.23	0.84

The textural properties namely specific surface area (A_spec_), micropore volume (V_Micro_) and external surface area (A_ext_) were determined using N_2_ physisorption (Fig. 1B, Table 2). According to these results, MOR contributes to the specific and external surface areas as well as micropore volume in DETOXSAN to a significantly higher extent compared to that of HEU. The lower specific surface area of HEU seems to be because of smaller pore size of the main channels of the medium-pore 10-membered ring zeolite^23^ compared to that of the large-pore 12-membered ring zeolite, MOR^24^. Therefore, the micropore volume of HEU can be blocked easily by inorganic salts residing inside the micropores or the physisorption probe molecule nitrogen is too large to enter the smaller pores. Compared to previous investigations^19^, the A_spec_ was higher even though the same pre-treatment to remove water from the zeolite pores (12 h at 250 °C under vacuum) was used. Compared to the Mexican zeolite, the A_spec_ of the Cuban one used for DETOXSAN was threefold higher.

**Table 2:** Textural properties of mordenite, clinoptilolite and DETOXSAN containing both zeolites shown textural properties are the specific surface area (A_spec_), external surface area (A_ext_), and micropore volume (V_micro_).

	Mordenite	Clinoptilolite	DETOXSAN
A_spec_/m^2^ g^−1^	497	48	165
A_ext_/m^2^ g^−1^	109	48	101
V_micro_/cm^3^ g^−1^	0.15	–	0.03

Similar to DETOXSAN, the reference HEU contains the same charge-compensating cations; however, with a significantly lower sodium cation content and a smaller Si/Al ratio of 5.7 (Table 1). The composition of natural zeolites, i.e., the type of cations associated with the aluminium centers within the framework depend on the conditions during their formation and show a high geochemical stability because of its natural origin^25^. Therefore, the Cuban zeolite in DETOXSAN contains more charge-compensating sodium ions than the natural Mexican one, which is the favored cation for ion exchange^14,26^. Sodium ions are weakly bound and rapidly released from the zeolite channels as reported for HEU^27^. Synthetic zeolites are conventionally synthesized in presence of one or two different types of cations depending on their application.

The synthetic MOR topology possesses only sodium as charge-compensating cation because of the composition of the synthesis gel. The Si/Al molar ratio of the microporous sample is 7.3. Like with HEU, the structure was identified by comparison with the simulated reference pattern from the ICSD^28,29^. The textural properties are listed in Table 2. This material is only comprised of zeolite MOR and does not contain any amorphous impurities. The nitrogen physisorption isotherm is similar to a type I(a) isotherm, which is characteristic of a microporous material with interparticle condensation^30^. The properties of both reference materials support previously published results and are required for evaluating the uptake and release of 5-HT-hc. Furthermore, it shows that the micropore volume observed for the Cuban zeolite can be predominantly attributed to the MOR fraction of the mixture.

According to studies performed with zeolite samples with a similar chemical composition as MOR and HEU, the decomposition of both crystalline frameworks can be neglected at 36 °C and a pH between 5 and 9^31,32^.

### Selecting a suitable 5-HT model molecule for uptake experiments

In preliminary investigations, the uptake of the pure amine 5-HT by the Cuban zeolite at two different pH values (5 and 7) and an incubation temperature of 36 °C was determined (Table 3). The same experimental setup described above was used for these 5-HT uptake studies, with the variation that larger amounts of solution were separated and filtrated for additional thermo-gravimetric analyses of the solid residue. Within 2 h at both pH levels, the 5-HT level decreased significantly. However, this observation cannot be related only to the adsorption of 5-HT on the zeolite, but rather to the degradation of the amine during incubation. Thus, 5-HT is a rather unstable molecule and not suitable for our experimental study. Outside a living organism, it is stored at 4 °C to prevent degradation^10^.

**Table 3:** 5-HT uptake capacity of DETOXSAN in relation to temperature and pH value.

Temperature/°C	pH	5-HT uptake (mg/g zeolite) at different incubation time intervals			
		15 min	30 min	60 min	120 min
36	5	33.8	21.9	15.4	8.3
36	7	19.2	15.2	9.8	8.4

5-HT uptake samples: 1 g of zeolite (particle size 40 µm), 100 ml of double distilled water (pH 7) or 0.00001 M HCl in 100 ml of double distilled water (pH 5), respectively, 25 mg of 5-HT, temperature = 36 °C. The TG measurements were conducted between 350 and 600 °C.

**Figure 2:**
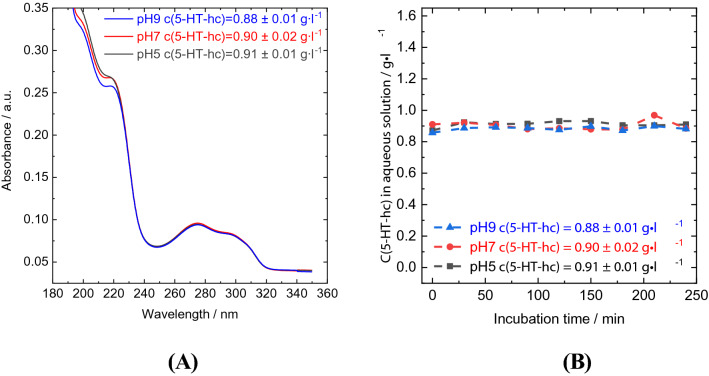
UV–Vis-spectra of 5-HT-hc at different pH values (**A**) and 5-HT-hc concentration over an incubation time of 4 h in the absence of a zeolite adsorbent (**B**).

For this reason, 5-HT-hc was chosen as the model compound. The UV–Vis spectra of 5-HT-hc at a pH of 5, 7, and 9 showed only some minor differences in intensity at ν < 240 nm, the characteristic bands were located at 198, 217, 275.5, and 296.5 nm (Fig. 2). Therefore, changes in the molecular structure at different pH values can be excluded. There was no change in concentration detected when the starting 5-HT-hc concentration was approximately 0.9 g l^−1^ and the sample was incubated for 4 h at 36 °C and a pH of 5, 7, and 9. Therefore, we concluded that the 5-HT-hc would not decompose during uptake and release studies and the changes in the liquid phase concentration are only caused by an interaction with the adsorbents.

### Adsorption of 5-HT-hc on DETOXSAN

**Figure 3:**
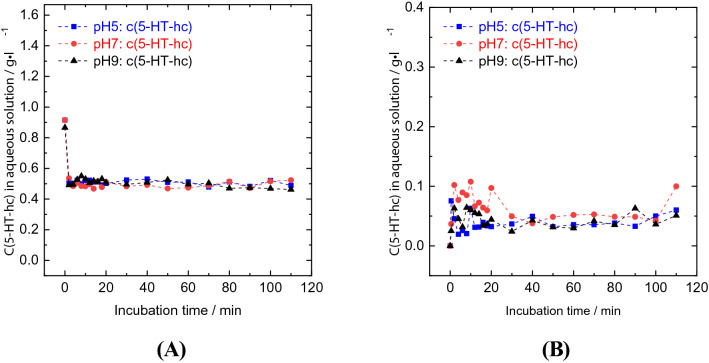
Uptake (**A**) and release (**B**) of 5-HT-hc by DETOXSAN at 36 °C and different pH levels.

The uptake of 5-HT-hc by DETOXSAN incubated for 110 min at a pH of 5, 7, and 9 is shown in Fig. 3A. In the first 2 min after the addition of DETOXSAN, we detected a steep decrease in the 5-HT-hc concentration within the liquid phase from an initial concentration of approximately 0.92 g l^−1^ to 0.50, 0.49, and 0.48 g l^−1^ at a pH of 5, 7, and 9, respectively. Thereafter, no further changes in the 5-HT-hc levels were observed regardless of the pH level; a steady state of 5-HT-hc uptake was reached after the first 2 min of incubation. Increasing the pH level from 5 to 7 resulted in a slight increase in 5-HT-hc uptake from 13.7 to 14.1 mg g^−1^. However, the uptake decreased by 1.5 mg g^−1^ when the pH was increased from 7 to 9 (Table 4). This indicates that the amount of 5-HT-hc bound to DETOXSAN is dependent on the concentration of protons in the solution. With a decreasing number of protons in the solution from pH 7 to 9 a possible deprotonation of 5-HT-hc hinders its uptake.

**Table 4:** 5-HT-hc uptake and release capacities of DETOXSAN at different pH values.

5-HT-hc adsorbed		5-HT-hc loading after the release experiment	
pH	mg g^−1^	pH	mg g^−1^
5	13.70	5	12.31
7	14.05	7	12.26
9	12.57	9	11.19

Amount of bound 5-HT-hc is averaged between 40 and 120 min after the addition of DETOXSAN for release and uptake studies, respectively. The fixed amine was calculated based on UV Vis data measured in aqueous solutions.

### Release studies of 5-HT-hc from DETOXSAN

After adding the loaded zeolites into aqueous solutions at different pH levels (5, 7, and 9) the 5-HT-hc released into the solution was measured using UV–Vis spectroscopy. The concentration profile of 5-HT-hc during the release study with DETOXSAN over an incubation time of 110 min has been shown in Fig. 3. About 2 min after the addition of loaded zeolite into the liquid phase, the concentration of 5-HT-hc reached 0.04, 0.05, and 0.04 g l^−1^ at a pH of 5, 7, and 9, respectively, without any further changes during the remaining incubation time. The amount of 5-HT-hc bound irreversibly by the zeolite changed only slightly from 12.31 mg g^−1^ at a pH of 5 to 12.26 mg g^−1^ at a pH of 7 up to 11.19 mg g^−1^ at a pH of 9 (Table 4). Therefore, most of the 5-HT-hc remained adsorbed on the zeolite independent of the proton concentration of the solution. Notably, the 5-HT-hc did not decompose during the uptake and release studies as shown in Fig. 2A. Moreover, it does not react with the active aluminium centers of the zeolite as elucidated by release studies that were independent of the pH value.

### Adsorption studies of 5-HT-hc on clinoptilolite and mordenite

We investigated the proportional effect of the two different zeolite types present in DETOXSAN on the uptake and release properties of 5-HT-hc. HEU and MOR were used in a similar experimental set-up as described above; however, only at 36 °C and a pH of 7. A steady state of amine uptake was reached after two minutes, thereby reducing the number of measurements at other time points (Fig. 3A). The loading of 5-HT-hc on the zeolite was calculated based on the remaining concentration of this amine in solution (Table 5). MOR with the larger micropore volume and external surface area exhibited a higher uptake of 5-HT-hc (16.7 mg g^−1^), whereas HEU with smaller micropores and specific surface area adsorbed less amines (15.0 mg g^−1^), which is comparable to DETOXSAN adsorption (14.1 mg g^−1^). Therefore, HEU, MOR and DETOXSAN exhibit a similar uptake capacity for 5-HT-hc, indicating that these materials interact with 5-HT-hc in a similar manner. Furthermore, no direct correlation seemed to exist between the amine uptake capacity and the specific and external surface areas as well as the micropore volume for these zeolites (Table 2).

**Table 5:** 5-HT-hc uptake and release capacities of clinoptilolite, mordenite and DETOXSAN at a pH of 7.

	Amount Adsorbed	Release study		
		Remaining	Released	
	mg g^−1^	mg g^−1^	mg g^−1^	%
Mordenite	16.66	15.39	1.30	7.60
Clinoptilolite	14.99	14.43	0.56	3.70
DETOXSAN	14.06	12.27	1.79	12.70

Amount of bound 5-HT-hc is the average value between 40 and 120 min after the addition of loaded zeolite samples into the incubation medium (see Fig. 5B).

**Figure 4:**
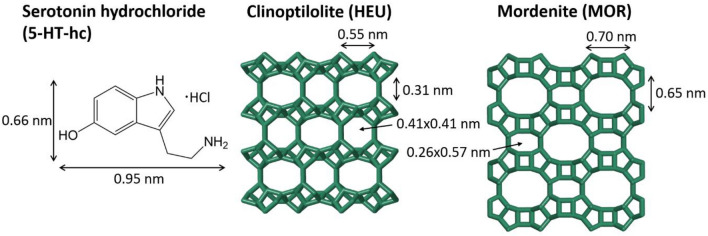
Structures of 5-HT-hc, clinoptilolite, and mordenite with the molecule and micropore dimensions. (5-HT-hc size from Chem3D by Perkin Elmer; zeolite pore dimensions from IZA homepage http://www.iza-structure.org/databases/*.*

This result is supported by the comparison between the dimensions of the micropores in HEU and MOR with that of 5-HT-hc. HEU micropores have dimensions of either 0.41 × 0.41 nm or 0.31 × 0.55 nm, while MOR topology has only one type of larger micropores with dimensions of 0.70 × 0.65 nm^27,30^, as shown in Fig. 4. The 5-HT molecule is significantly larger than the micropore sizes in these frameworks, making the entry of 5-HT into the zeolite pores highly unlikely. Therefore, the adsorption of 5-HT-hc can occur only on the external zeolite surface. This is in line with the fast adsorption of this molecules observed within a few minutes, indicating an absence of mass transfer limitations that would apply within the narrow micropores. The UV–Vis spectra of 5-HT-hc in the liquid phase, shown in Fig. 6, were recorded 40 min after the addition of the zeolites. No change in position and width of the adsorption bands was observed, indicating that there were no changes in the molecular structure of 5-HT-hc, regardless of the zeolite.

### Release studies of 5-HT-hc from clinoptilolite and mordenite

**Figure 5:**
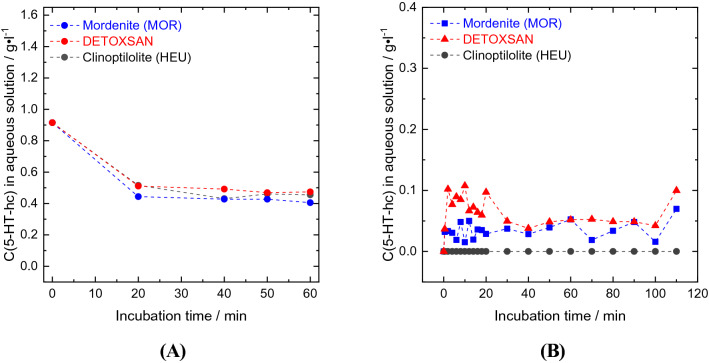
Uptake (**A**) and release study (**B**) of 5-HT-hc at 36 °C and a pH of 7 by different zeolites and DETOXSAN.

**Figure 6:**
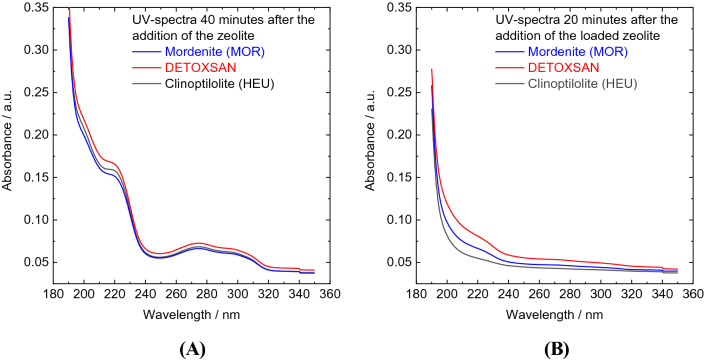
UV–Vis spectra of 5-HT-hc in the liquid phase (**A**) 40 min after the addition of the respective zeolite and (**B**) 20 min after the addition of the respective loaded zeolite into the liquid phase.

In order to gain further insight into how DETOXSAN and its active components, MOR and HEU, interact with 5-HT-hc, both pure zeolites were used for release studies at a pH of 7, as shown in Fig. 5. After adding loaded MOR to deionized water, a stable concentration of 0.04 g l^−1^ 5-HT-hc was determined within the liquid phase, implying that more than 90% (15.39 mg g^−1^) of the 5-HT-hc remain bound to the zeolite surface (Table 5). HEU released less, whereas DETOXSAN released more 5-HT-hc into the liquid phase. This is also visible on the UV–Vis spectra shown in Fig. 6B, where no characteristic bands for 5-HT-hc were observed during the first 20 min in the release study with HEU compared to those for MOR and DETOXSAN. No major changes in the UV–Vis spectra were observed for these minerals even after 20 min. This implies that an interaction between the 5-HT-hc and the zeolite framework is taking place and that this physical interaction depends on the framework topology. In contrast to previously described chemical and textural properties (Table 2), HEU with the lowest specific surface area and accessible micropore volume, took up 5-HT-hc only slightly lesser than that by MOR and released it only slightly slower under the investigated conditions. Therefore, the uptake and release capacity seem to be independent of the micropore volume. A lower Si/Al ratio of HEU (Table 1) results in a higher hydrophilicity and higher basicity of the overall framework^33^; however, this parameter is unable to clarify the kind of interaction between 5-HT-hc and the crystal lattice. Overall, we concluded that the adsorption of 5-HT-hc by all three zeolites is high and the fixed amine is hardly released with rather small differences among the different zeolite types.

The adsorption of 5-HT-hc by zeolites differs only in some aspect from the linkage of histamine, another essential native amine involved in the human physiology. In addition to the amine group, 5-HT also possess a hydroxyl group, making its molecular weight 176, which is larger and heavier than histamine, an imidazolyl derivative with a molecular weight of 111. Both amines are adsorbed by the zeolites to a similar degree; however, DETOXSAN binds about 35 wt% more histamine than HEU^19^, whereas HEU binds about 6 wt% more 5-HT-hc than DETOXSAN. The reason for this difference is currently unknown. There are no correlations between the adsorption capacity and the external as well as internal surface areas for both amines. Although there seem to be no big differences in 5-HT adsorption by the zeolites, their eligibility to be described for oral consumption needs to be carefully checked^14^.

Finally, the reason behind scientifically understanding the loading capacity of histamine and 5-HT by zeolites is important, even though they are currently used as a medical intervention. The oral application of zeolite causes them to be loaded by histamine within the stomach which plays an important role in gastric acid production^34^. However, the therapeutical aim is to reduce 5-HT level within the bowels, thereby reducing diarrhea, as shown in carcinoid patients with a responder rate of about 70%^8^. It is essential to investigate whether enough binding sites for 5-HT still exist at zeolite surface or whether they have already been occupied by histamine during passage through the stomach. Therefore, whether an additive or competitive linkage of both amines with the framework occurs is the deciding factor in selecting the type of formulation. This means that ascertaining whether orally applied zeolite is still available in the gut or not before the gut might prompt the use of enteric-coated capsules. Future investigations are required to make such decisions.

## Methods

### Materials

The naturally occurring zeolites used in this study are the same as reported previously^18,19^ and were obtained from Cuba, San Andrés and Mina San Francisco, San Felipe, Guanajuato in Mexico. The zeolites were used as received from the respective supplier without any further chemical or thermal treatment. The zeolite sample from Mexico was sieved and the fraction below 40 µm was used in this study. This corresponded to the particle size of the Cuban zeolite, which was about 40 µm. The Mexican sample contains 65 wt% HEU (three letter code assigned by the international zeolite association (IZA) for clinoptilolite) 35 wt% of amorphous non-identified material, while the Cuban zeolite consists of 43 wt% HEU, 35 wt% MOR and 22% non-identified materials as described^19^. As a comparative sample MOR was synthesized following the recipe by Kim et al.^35^. 5-HT-hc had a purity of 98% and was supplied by Alfa Aesar. Sodium hydroxide pellets and concentrated hydrochloric acid were acquired from VWR Chemicals.

### Uptake and release studies

All uptake and release studies were performed in 100 ml three-neck flasks. The temperature was regulated using a thermocouple in an external water bath, which was continuously stirred with a magnetic stirring bar at 300 rpm. The temperature inside the flask was monitored using a mercury thermometer. To achieve a homogenous suspension, an additional magnetic stirring bar was placed inside the flask. Binding capacities for 5-HT-hc were tested at a pH of 5, 7, and 9. The experiments at a pH 7 were performed in pure distilled water, whereas pH 5 and 9 were adjusted with 0.01 M aqueous hydrochloric acid (HCl) and sodium hydroxide (NaOH), respectively. The pH levels were monitored using a pH sensor GMH 3530 manufactured by Greisinger Electronic GmbH, Regenstauf, Germany, which was calibrated before use with a three-point calibration. A volume of 100 ml of the forementioned test solutions was placed in the three-neck flask and the temperature was adjusted to 36 °C. Then, 5-HT-hc was added to obtain a concentration of around 0.9 g l^−1^ in the liquid phase after its complete dissolution, and the zeolite adsorbent was added to obtain a concentration of 30 g_zeolite_ l^−1^, independent of the chosen sample. After a certain time, the suspension was filtered using a Büchner funnel and checked for 5-HT-hc levels. For the release studies, the loaded zeolite was added again to 100 ml of the solution with the desired pH level. The 5-HT-hc concentration in the solution was monitored using UV–Vis spectroscopy until no further quantitative changes occurred.

### UV–Vis spectroscopy

In order to monitor the 5-HT-hc concentration within the liquid phase for the uptake and release experiments, small aliquots (1.5 ml) were taken using a syringe equipped with a filter having a pore width of 0.4 µm. The samples taken were diluted by a factor of 500 to obtain a solution, where the extinction detected by UV–Vis spectroscopy is directly proportional to the concentration of 5-HT-hc. Calibration curves are shown in Fig. S3. The 5-HT-hc concentration was measured employing a JASCO UV–Vis spectrometer V-650 with a wavelength range between 190 and 850 nm and an increment of 0.5 nm. Characteristic UV–Vis spectra of 5-HT-hc are shown in Fig. 2.

### Thermogravimetric analysis

For a description of adsorbed 5-HT on DETOXSAN, thermogravimetric analysis was used as previously described^14^. Therefore, the samples loaded with 5-HT were analyzed by TG–DTA analysis (TA instruments SDT 2960). Analogous to previously published studies^19^, the difference in weight loss between the 5-HT-loaded zeolite and the unloaded zeolite in a temperature range of 350–600 °C was considered for 5-HT uptake by the respective samples. The samples were heated up at a rate of 10 K min^−1^ from room temperature to 700 °C under air.

### Powder X-ray diffraction

The powder X-ray diffraction (PXRD) patterns of the zeolite samples were recorded on a Philipps 15 X’pert Pro X-ray diffractometer using a variable blend and Cu-Kα radiation. The XRD patterns were collected in the 2θ range from 2° to 50° with a step size of 0.017°.

### Nitrogen physisorption

Nitrogen physisorption isotherms were recorded at 77 K using a Quantachrome Autosorb-1 instrument. Prior to the measurements, the samples were outgassed for 12 h at 250 °C under vacuum until a pressure of 0.04 × 10^–3^ torr was reached. Micropore volume and external surface area were determined through the t-plot method using non-porous silica as reference material based on IUPAC recommendations^30^.

### ICP-OES

The chemical composition of the zeolite samples was determined by ICP-OES using a Spectro Analytical Instruments instrument (Model: CIROS^CCD^). The solid-state samples (0.1 g) were dissolved in 4 ml of 37 wt% HCl, 2 ml of 65 wt% HNO_3_, and 8 ml of 40 wt% HF and digested in a microwave.

## Supplementary Information

Supplementary Information.

## Abbreviations

5-HT: Serotonin
5-HT-hc: Serotonin hydrochloride
HEU: Clinoptilolite
MOR: Mordenite
ICP-OES: Inductively coupled plasma-optical emission spectrometry
