# Supplementary material for: Uptake and release characteristics of serotonin hydrochloride by natural Cuban zeolite containing clinoptilolite and mordenite
Source: Sci Rep. 2021 Jul 12;11:14277. doi: 10.1038/s41598-021-93487-z (PMC8275685; doi:10.1038/s41598-021-93487-z)
Supplement: Supplementary file 1 — Supplementary Information. [file 41598_2021_93487_MOESM1_ESM.docx]

**Uptake and release characteristics of serotonin hydrochloride by natural Cuban zeolite containing clinoptilolite and mordenite**

*Scientific Reports*

Jan-Paul Grass, Ulrike Pals, Alexandra Inayat, Wilhelm Schwieger, Martin Hartmann, Wilfried Dathe*

# ^*^Corresponding author e-mail: daweidoc@gmx.de

# Supplementary Fig. S1 - Diffraction patterns of Cuban zeolite containing mordenite and clinoptilolite, with simulated XRD patterns with the respective ICSD code [24, 25].

**Supplementary Fig. S2** - Diffraction patterns of pure clinoptilolite and mordenite with simulated reference patterns with their respective ICSD code [24, 25].

**Supplementary Fig. S3 –** Calibration curve from UV-Vis spectra of 5-HT-hc with a dilution of 500 and the corresponding calibration curve for the diluted concentrations.
